# Untargeted Chemical Profile, Antioxidant, and Enzyme Inhibition Activity of *Physalis angulata* L. from the Peruvian Amazon: A Contribution to the Validation of Its Pharmacological Potential

**DOI:** 10.3390/antiox14030246

**Published:** 2025-02-20

**Authors:** Gabriel Vargas-Arana, Alfredo Torres-Benítez, José Erick Ortega-Valencia, Claudia Merino-Zegarra, Pilar Carranza-Rosales, Mario J. Simirgiotis

**Affiliations:** 1 Laboratorio de Química de Productos Naturales, Instituto de Investigaciones de la Amazonía Peruana, Avenue Abelardo Quiñones Km 2.5, Iquitos 16001, Peru; cmerino@iiap.gob.pe; 2 Facultad de Industrias Alimentarias, Universidad Nacional de la Amazonía Peruana, Iquitos 16001, Peru; 3 Carrera de Química y Farmacia, Facultad de Ciencias, Universidad San Sebastián, General Lagos 1163, Valdivia 5090000, Chile; alfredo.torres@uss.cl; 4 Tecnológico Nacional de México, Instituto Tecnológico Superior de Xalapa, Sección 5ª Reserva Territorial S/N, Col. Santa Bárbara 91096, Veracruz, Mexico; erick.ortega@itsx.edu.mx; 5 Centro de Investigación Biomédica del Noreste, Instituto Mexicano del Seguro Social, Monterrey 64720, Nuevo León, Mexico; carranza60@yahoo.com.mx; 6 Instituto de Farmacia, Facultad de Ciencias, Universidad Austral de Chile, Campus Isla Teja, Valdivia 5090000, Chile; mario.simirgiotis@uach.cl

**Keywords:** *Physalis angulata*, extracts, bioactive compounds, biological activity, therapeutic properties

## Abstract

*Physalis angulata* is a plant of great value in traditional medicine known for its content of bioactive compounds, such as physalins and withanolides, which possess diverse biological activities. In this study, the chemical profile, antioxidant activity, and enzyme inhibition capacity of aqueous and ethanolic extracts obtained from the root, stem, leaves, calyx, and fruits of *P. angulata* collected in Peru were evaluated. A total of forty-two compounds were detected in the extracts using UHPLC-ESI-QTOF-MS analysis. In vitro analyses revealed that leaf extracts contained the highest concentration of phenolic compounds, while leaf and fruit extracts showed the best results in FRAP, DPPH, and ABTS antioxidant tests; on the other hand, inhibition of AChE, BChE, α-glucosidase, and α-amylase enzymes was variable, but calyx and fruit extracts showed higher effectiveness. In silico analyses indicated that the compounds physagulin A, physagulin F, physagulide P, physalin B, and withaminimin showed stable interactions and favorable binding affinities with the catalytic sites of the enzymes studied. These results confirm the pharmacological potential of extracts and compounds derived from different organs of *P. angulata*, suggesting their promising use in treating diseases related to the central nervous system and metabolic syndrome.

## 1. Introduction

**Figure 1:**
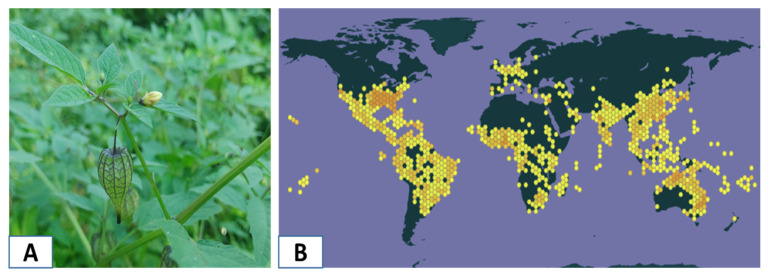
(**A**) Bolsa mullaca plant (*Physalis angulata*). (**B**) Distribution of *Physalis angulata* (GBIF).

*Physalis angulata* is classified as an herbaceous plant belonging to the family Solanaceae. It grows up to 1600 m above sea level, and its distribution ranges from the United States to Argentina, but it is naturalized in most of the world. Botanically, it is characterized by erect, angular stems with trichomes, ovate-lanceolate leaves, flowers with pedicels, a sub-conical calyx, a rotate corolla, and a berry fruit (Figure 1) [1,2]. In various parts of the world, this species has been widely used in traditional medicine for the treatment of infections, inflammations, and metabolic disorders. In the case of Peru, *P. angulata* is recognized as a medicinal and culturally valuable plant resource, mainly used to manage diseases such as diabetes, nervous system disorders, gastrointestinal conditions, and asthma [3], and for its potential in various biotechnological applications [4,5].

In *P. angulata*, multiple phytochemical constituents have been reported, among which physalins [6] and withanolides [7] predominate, as well as flavonoids, terpenes, carotenoids, and new compounds from these families that continue to be isolated and purified that complement the pharmacological properties of the species [8,9,10,11,12,13]. Moreover, this is in addition to the studies on *Physalis* species highlighting the evaluation of immunomodulatory signaling and anti-inflammatory activity in different cellular and animal models [14,15,16,17,18,19]. In addition, withanolides present in the species have shown cytotoxic effects on different cancer cell lines, including retinoblastoma [20] and melanoma [21], as well as a high potential in the management of autoimmune diseases, such as scleroderma, by modulating inflammatory and fibrogenic biomarkers [22].

At the biochemical and molecular level, key genes in the biosynthesis of withanolides and other bioactive compounds have been identified by transcriptomic analysis and RNA interference assays (RNAi) [23]. Likewise, metabolomic studies have characterized the phytochemical profiles of the plant in response to different stimuli, such as methyl jasmonate, highlighting its ability to induce the accumulation of metabolites with pharmacological potential [24]. It is also important to highlight the ecological relationships that exist between *P. angulata* and species of fungi and lepidoptera, which can influence the production of secondary metabolites and their biological effects [25,26].

In parallel, genomic studies have provided valuable insights into the organization of the plastome and the phylogenetic relationships within the genus *Physalis*, shedding light on its evolutionary history and patterns of diversification [27,28,29]. These studies have revealed structural variations, gene content, and adaptive mechanisms that contribute to the ecological success and metabolic versatility of the plants. In addition, the identification of key genes involved in the biosynthesis of bioactive compounds has opened new avenues for biotechnological applications, particularly in the pharmaceutical industry [30]. These genetic discoveries facilitate the targeted production of withanolides and other pharmacologically relevant metabolites, potentially leading to the development of novel therapeutic agents [31].

In this study, the chemical characterization of aqueous and ethanolic extracts from different organs of *P. angulata* (root, stem, leaves, calyx, and fruits) collected in the Peruvian Amazon was addressed. Additionally, their biological activities were evaluated, focusing on antioxidant properties and enzyme inhibitory activities through in vitro and in silico methods.

## 2. Materials and Methods

### 2.1. Plant Material

Leaves, stem, fruits, calyces, and roots of *Physalis angulata* were collected in October 2022, near the town of Mohena Caño, on the road to Canta Gallo (03°46′14.9″ S y 73°14′27.0″ W), Belen district, Maynas province, Loreto region, Peru. The plant was identified in the Herbarium Amazonense of the National University of the Peruvian Amazon; a voucher specimen was archived with the identification number 9491. 

### 2.2. Preparation of Extracts

Leaves, stem, calyces, and roots were oven-dried at 40 °C for a duration of 48 h and then were homogenized using a blade mill into powder. The fruits were first homogenized and then freeze-dried at −55 °C and 0.021 mBar for 48 h. Two types of extracts were prepared: one ethanolic and one aqueous. For each case, 20 g of dry and ground sample and 200 mL of solvent were used. The ethanolic extract was prepared by maceration for a period of 48 h, then filtered and concentrated under reduced pressure at 40 °C. For the aqueous extract, boiled water was added, allowed to reach room temperature, then filtered and freeze-dried.

### 2.3. LC Parameters and MS Parameters

The identification and separation of compounds in the extracts were conducted using a UHPLC-ESI-QTOF-MS system, which included a UHPLC Ultimate 3000 RS with Chromeleon 6.8 software (Dionex GmbH, Idstein, Germany) and a Bruker maXis ESI-QTOF-MS operated with the Data Analysis 4.0 software (Bruker Daltonik GmbH, Bremen, Germany). For analysis, 5 mg of each extract was dissolved in 2 mL of methanol, filtered through a polytetrafluoroethylene (PTFE) membrane, and 10 µL was injected into the system. The chromatographic setup featured a quaternary pump, an autosampler, a thermostated column compartment, and a photodiode array detector. A binary gradient system was used for elution, with eluent (A) consisting of 0.1% formic acid in water and eluent (B) containing 0.1% formic acid in acetonitrile. The gradient profile was as follows: 12% B isocratic (0–1 min), 12–99% B (2–15 min), 99% B isocratic (16–18 min), 99–12% B (18–18.20 min), and 12% B (18.2–20 min). The separation was performed on a Thermo 5 µm C18 80 Å column (150 mm × 4.6 mm) at a flow rate of 0.3 mL/min. The ESI-QTOF-MS analyses were carried out in negative ion mode, covering a scan range of 100–1200 *m*/*z*. The electrospray ionization (ESI) parameters included a capillary temperature of 200 °C, a capillary voltage of 2.0 kV, a dry gas flow rate of 8 L/min, and a nebulizer pressure of 2 bar. All experiments were conducted in automatic MS/MS mode, and the structural characterization of secondary metabolites was based on high-resolution full MS, fragmentation patterns, and comparisons with the literature.

### 2.4. LC Total Phenolic (TP)

The total phenolic content was determined using a colorimetric method adapted from Velioglu et al. [32] with slight modifications. A 100 µL aliquot of the extract (2 mg/mL) was combined with 750 µL of Folin–Ciocalteu reagent, previously diluted (1:10) with Milli-Q water. After incubating in the dark for 5 min, 750 µL of sodium bicarbonate solution (60 g/L) was added. The mixture was then kept in the dark at 30 °C for 90 min, after which absorbance was measured at 725 nm using a Cary60 UV–visible spectrophotometer. A standard curve was generated using gallic acid (10–100 μg) and the results were expressed as mg of gallic acid per gram of extract.

### 2.5. Antioxidant Activity

#### 2.5.1. DPPH Scavenging Activity

The DPPH• (2,2-diphenyl-1-picrylhydrazyl) radical was assayed by the decolorization method [33]. A 3.9 mL aliquot of a DPPH• radical solution (100 μM) prepared in 80% methanol was mixed with 0.1 mL of the extract (2 mg/mL), which had been previously filtered through a 0.45 μm membrane filter. The mixture was stirred vigorously and incubated in the dark at 25 °C for 30 min. After incubation, absorbance was measured at 517 nm using a Cary60 UV–visible spectrophotometer. The DPPH• concentration in the reaction medium was determined from a calibration curve using linear regression. The control sample contained 0.1 mL of 80% aqueous methanol and 3.9 mL of DPPH• solution (100 µM). Results were expressed as the Trolox equivalent antioxidant capacity (TEAC) in μmol Trolox/g of extract. The synthetic antioxidant Trolox, at concentrations ranging from 5 to 30 µM in 80% methanol, was used as a reference under the same conditions.

#### 2.5.2. ABTS Bleaching Capacity

The ABTS (2,2′-azino-bis(3-ethylbenzothiazoline-6-sulfonic acid)) assay was performed by bleaching of the cationic radical ABTS•+ as described by Re et al. [34]. The reaction was initiated by adding 1500 μL of an ABTS•+ solution in PBS buffer (with an initial absorbance of 0.70 ± 0.02 at 734 nm) to 500 μL of the extract (2 mg/mL) in a cuvette maintained at 30 °C. The mixture was homogenized and allowed to react for 7 min, after which the absorbance was measured at 734 nm using a Cary60 UV–visible spectrophotometer. Results were expressed as the Trolox equivalent antioxidant capacity (TEAC) in μmol Trolox/g of extract. A calibration curve for the TEAC was established using Trolox solutions at concentrations ranging from 4 to 14 µM in PBS buffer under the same conditions.

#### 2.5.3. Ferric-Reducing Antioxidant Power Assay (FRAP)

The FRAP was conducted following the Benzie and Strain method [35]. A 10 μL aliquot of the extract (2 mg/mL) was combined with 90 μL of distilled water and 900 μL of the FRAP reagent, which consisted of 2.5 mL of 10 μM 2,4,6-tripyridyl-s-triazine solution in 40 mM HCl, 2.5 mL of 20 μM FeCl_3_, and 25 mL of 0.3 μM acetate buffer at pH 3.6. After allowing the reaction to proceed for 7 min, absorbance was measured at 593 nm using a Cary60 UV–visible spectrophotometer. The results were expressed as the Trolox equivalent antioxidant capacity (TEAC) in μmol Trolox/g of extract.

### 2.6. Enzymatic Inhibitory Activity

#### 2.6.1. Cholinesterase Inhibition Assay

The galantamine standard was prepared at concentrations between 0.05 and 25 µg/mL to make the calibration curve. In a 96-well microplate, 100 µL of 3 mM DTNB (solution in 50 mM Tris-HCl buffer at pH 8.0 with 0.1 M NaCl and 0.02 M MgCl_2_) was added, then 20 µL of acetylcholinesterase or butyrylcholinesterase (0.26 U/mL) dissolved in Tris-HCl buffer at pH 8.0, 20 µL of positive control, the extract sample (1 mg/mL) or blank, and 40 µL of the same buffer, and allowed to incubate for 15 min at 25 °C in the dark; subsequently, the reaction was initiated with the addition of 20 µL of acetylthiocholine iodide (15 mM) or butyrylthiocholine chloride (15 mM), as appropriate for the assay. The absorbances of the samples, positive control (Galantamine), and blank were recorded at 405 nm every minute for 30 min at 25 °C. Values were expressed as IC_50_ values (µg extract/mL) [36].

#### 2.6.2. α-Glucosidase Inhibition Assay

Extract solutions were combined with a sodium phosphate buffer and α-glucosidase, and then incubated at 37 °C for 15 min. Subsequently, p-nitrophenyl-α-d-glucopyranoside was added, and the mixture was further incubated for 30 min at 37 °C. Absorbance was then recorded at 415 nm using a microplate reader (BioTek Instrument, Inc., Winooski, VT, USA). Results were expressed as IC_50_ values (µg extract/mL), with acarbose serving as the positive control [37].

#### 2.6.3. α-Amylase Inhibition Assay

To the solutions of each extract was added deionized water, 1% starch solution, α-amylase, and it was incubated for 10 min at 37 °C. Subsequently, 200 μL of the mixture was taken and 100 μL of DNS (3,5-dinitrosalicylic acid) reagent solution was added, and the new mixture was taken to a thermoregulated bath at 85 °C for 30 min and allowed to cool. Subsequently, 900 μL of deionized water was added, and absorbance was measured at 515 nm using a microplate reader (BioTek Instrument, Inc., Winooski, VT, USA). Results were expressed as IC_50_ values (µg extract/mL), with acarbose serving as the positive control [38].

### 2.7. Calculation of ADME Parameters

The ADME (absorption, distribution, metabolism, and excretion) pharmacokinetic properties of the identified compounds from *Physalis angulata* were evaluated with the computational tool Osiris Data Warrior (v 5.5.0) to see which compounds are favorable as inhibitors of acetylcholinesterase (TcAChE), butyrylcholinesterase (hBChE), α-amylase, and α-glucosidase. The molecular descriptors that were calculated were the logarithm of the partition coefficient (cLogP), which must be <5, the number of hydrogen bond acceptors < 10, molecular mass of compounds < 500 Da, the number of rotatable bonds < 10, the number of hydrogen bond donors < 10, and the violations of Lipinski’s rule of five < 1 [39,40].

### 2.8. Calculation of Risk Toxicity

An in silico analysis of the toxicological behavior of the compounds identified in *Physalis angulata* was carried out. For this analysis, the Osiris Data Warrior computational tool was employed to evaluate potential risks, including mutagenicity, tumorigenicity, irritation, and reproductive effects [39,41]. Compounds that presented at least a risk of toxicity were discarded for the molecular docking analysis on the enzymes to be evaluated.

### 2.9. In Silico Analysis

To perform the in silico analysis of the compounds obtained from *Physalis angulata*, only those with no more than one violation in the pharmacokinetic evaluation and no detected toxicological risk were selected. The two-dimensional structures of the selected compounds were generated using ChemDraw 8.0 (PerkinElmer Informatics, Waltham, MA, USA) and then imported into the Avogadro software 1.2.0 (https://avogadro.cc, accessed 26 April 2024) for geometric optimization using the MMFF94 force field [41,42]. After energy minimization, the optimized structures were saved in mol2 format for molecular docking studies targeting acetylcholinesterase (TcAChE), butyrylcholinesterase (hBChE), α-amylase, and α-glucosidase. Galantamine served as the reference inhibitor for TcAChE and hBChE, while acarbose was used for α-amylase and α-glucosidase [43].

The crystallographic structures of these enzymes—*Torpedo californica* acetylcholinesterase (TcAChE; PDBID: 1DX6), human butyrylcholinesterase (hBChE; PDBID: 4BDS), α-amylase (PDBID: 2QV4), and α-glucosidase (maltase; PDBID: 2QMJ)—were retrieved from the RCSB PDB protein database (https://www.rcsb.org/ligand/APB, accessed 26 April 2024). These structures were crystallized with their respective reference inhibitors, galantamine for TcAChE and hBChE, and acarbose for α-amylase and α-glucosidase [43,44]. Enzyme preparation was carried out using the UCSF Chimera software (v1.16, San Francisco, CA, USA), where water molecules and co-crystallized ligands were removed from the active sites. Additionally, polar hydrogen atoms were added at pH 7.4, and appropriate ionization states for basic and acidic residues were assigned [42,45].

Molecular docking simulations were performed using rigid protein structures and flexible ligands, with torsion angles identified for each compound (10 independent runs per ligand). The docking process was conducted by targeting the catalytic pocket of the reference inhibitors, galantamine for TcAChE and hBChE, and acarbose for α-amylase and α-glucosidase. The docking grid was created using Autodock Vina in UCSF Chimera (Table 1). Results were analyzed and visualized with the Discovery Studio Visualizer software version 4.5 [46], focusing on the best ligand conformations for hydrogen bonding and π interactions, as well as the binding energy values (Kcal/mol) [39,41].

### 2.10. Statistical Analysis

All experiments were conducted in triplicate and the results were presented as mean ± standard deviation (SD) using Sigma Plot 11.0. Statistical comparisons were performed through one-way analysis of variance (ANOVA), followed by Tukey’s honestly significant difference (HSD) test, considering a significance level of *p* < 0.05.

## 3. Results

### 3.1. Untargeted Chemical Profile of P. angulata Extracts

**Figure 2:**
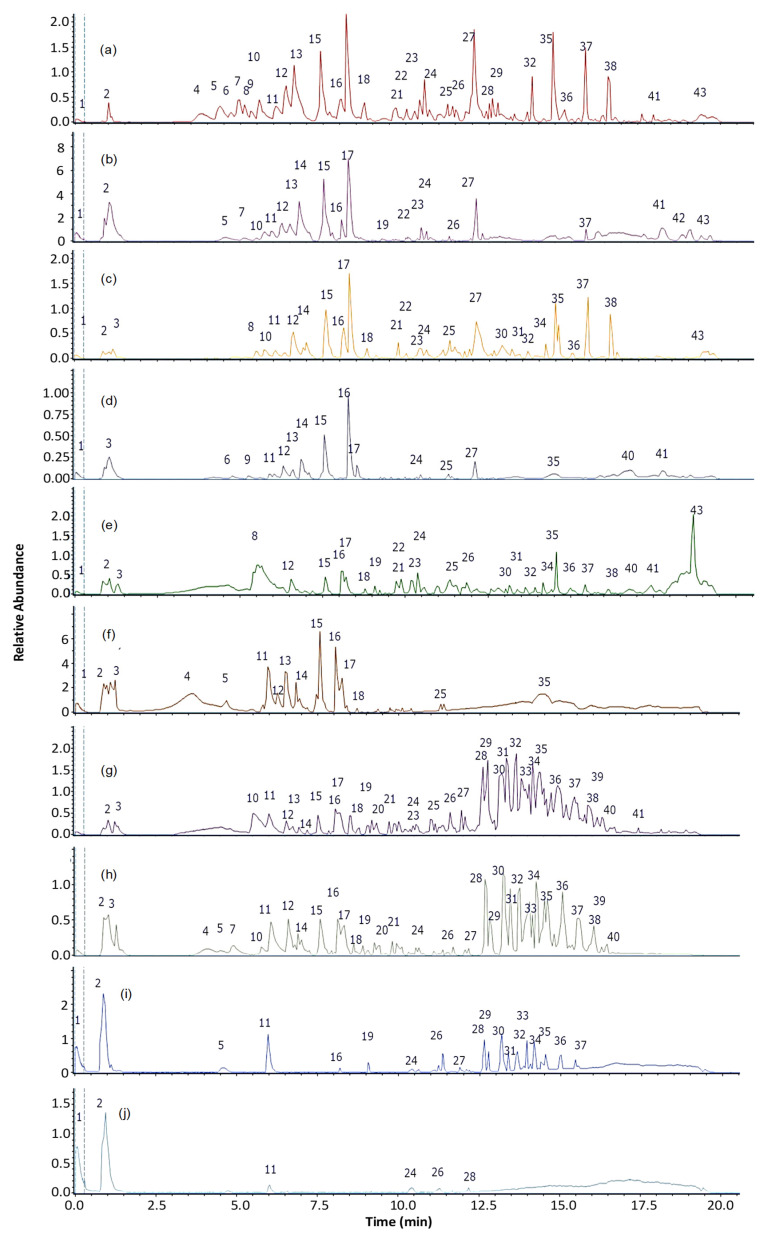
UHPLC-MS chromatograms: (**a**) ethanolic and (**b**) aqueous root extract; (**c**) ethanolic and (**d**) aqueous stem extract; (**e**) ethanolic and (**f**) aqueous leaf extract; (**g**) ethanolic and (**h**) aqueous calli extract; (**i**) ethanolic and (**j**) aqueous fruit extract.

Using a non-targeted metabolomic approach with high-resolution mass spectrometry (UHPLC-MS) in non-negative mode, the chemical composition of ethanolic and aqueous extracts of root, stem, leaves, calyx, and fruits of *P. angulata* was evaluated. A total of 42 compounds (Figure 2), including flavonoids, organic acids, and withanolides especially, were detected (Table 2). The retention times ranged from 0.45 min to 19.23 min, with different UV absorbance maxima that facilitated the identification of the compounds based on their spectral profile. Additionally, most compounds exhibited a mass accuracy below 5 ppm, confirming the high precision and reliability of the data obtained from the chromatographic method.

Phenolic acids: Twelve phenolic acids were tentatively identified in the peaks 2, 3, 4, 5, 6, 7, 8, 9, 10, 13, 14, and 17 as p-coumaric acid, gluconic acid, malic acid, citric acid, quinic acid, 3,4-dihydroxybenzoic acid, 2-(8-hydroxyoctyl)-6-methoxybenzoic acid, feruloyltyramine, 3-O-caffeoylquinic acid, ferulic acid, 3-O-p-coumaroylquinic acid, and 5-O-caffeoylquinic acid (C_9_H_8_O_3_, C_6_H_12_O_7_, C_4_H_6_O_5_, C_6_H_8_O_7_, C_7_H_12_O_6_, C_7_H_6_O_4_, C_16_H_24_O_4_, C_18_H_19_NO_4_, C_16_H_17_O_9_−, C_10_H_10_O_4_, C_16_H_18_O_8_ and C_16_H_18_O_9_), respectively.

Flavonoids: Four flavonoids were tentatively identified as scaposin (C_19_H_18_O_9_, pick 15), nevadensin (C_18_H_16_O_7_, peak 16), quercetin (C_15_H_10_O_7_, peak 41), and isorhamnetin (C_16_H_12_O_7_, peak 42).

Diterpenoids: Two diterpenoids were identified tentatively identified as forskolin (C_22_H_34_O_7_, peak 11, ion at *m*/*z*: 410.22992) and sclareol (C_20_H_36_O_2_, peak 43, ion at *m*/*z*: 308.27115).

Withanolides: Thirteen withanolides were tentatively identified in the peaks 12, 18, 20, 21, 22, 23, 26, 27, 29, 30, 32, 33, and 40 as physalin B, physalin A, physagulin I, physagulin F, physangulidine B, physalin D, physagulide P, withangulatin A, Withaminimin, physangulidine A, physangulidine C, physagulin A, and physangulidine C (C_27_H_30_O_15_, C_28_H_30_O_10_, C_30_H_39_ClO_8_, C_30_H_40_O_9_, C_28_H_36_O_8_, C_28_H_32_O_11_, C_30_H_40_O_9_, C_30_H_38_O_8_, C_30_H_40_O_8_, C_28_H_36_O_8_, C_28_H_35_O_8_, C_30_H_38_O_7_ and C_18_H_22_O_5_), respectively.

Fatty acids: Eleven fatty acids were identified tentatively identified as 2-hydroxy-3-methylpentanoic acid (C_6_H_12_O_3_, peak 19), eicosanoic acid (C_20_H_40_O_2_, peak 24), myristic acid (C_14_H_28_O_2_, peak 25), arachidonic acid (C_20_H_32_O_2_, peak 28), pygenic acid (C_30_H_48_O_4_, peak 31), (R)-2-hydroxystearic acid (C_18_H_36_O_3_, peak 34), N-oleoyl-phenylalanine (C_27_H_43_NO_3_, peak 35), N-oleyl-leucine (C_24_H_45_NO_3_, peak 36), heneicosanoic acid (C_21_H_42_O_2_, peak 37), tricosanoic acid (C_23_H_46_O_2,_ peak 38), and behenic acid (C_22_H_44_O_2_, peak 39).

### 3.2. Total Phenolic Contents and Antioxidant Activity

Table 3 presents the results of the antioxidant tests for the total phenolic content (TPC), DPPH, ABTS, and FRAP obtained from aqueous and ethanolic extracts of five organs of *P. angulata* (root, stem, leaves, calyx, and fruits).

### 3.3. Enzyme Inhibition Activity

Table 4 presents the results of the enzyme inhibition analyses for acetylcholinesterase (AChE), butyrylcholinesterase (BChE), α-glucosidase, and α-amylase obtained from the aqueous and ethanolic extracts of five *P. angulata* organs (root, stem, leaves, calyx, and fruits).

### 3.4. ADME Prediction

**Figure 3:**
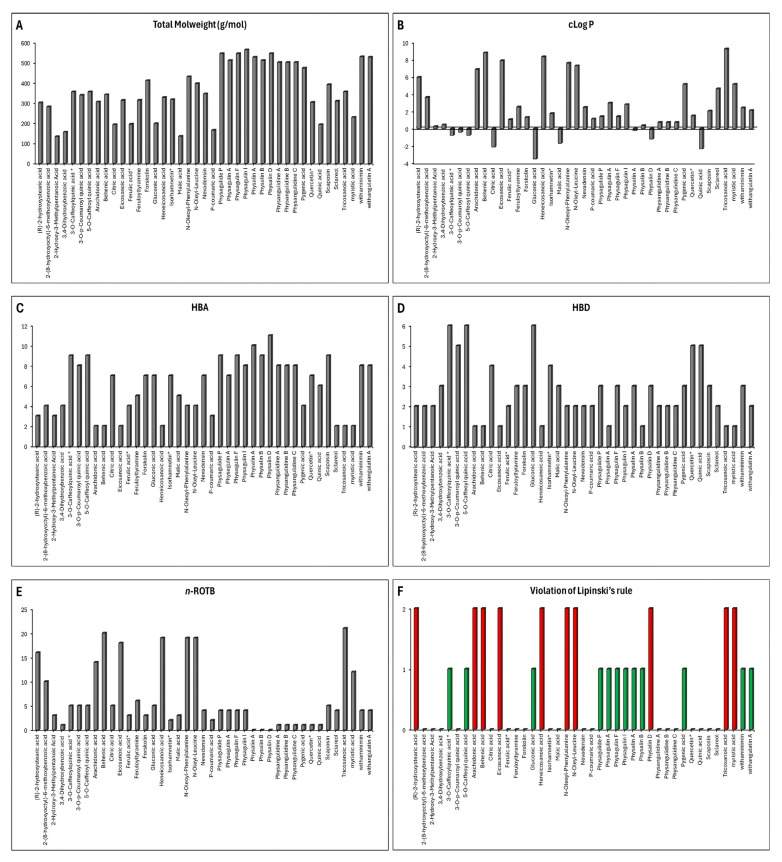
Pharmacokinetic properties of compounds obtained from *Physalis angulata* calculated with Osiris Data Warrior software. Note: red color indicates that it does not comply with pharmacokinetic analysis and green color indicates that it complies with pharmacokinetic analysis.

The compounds extracted from *Physalis angulata* were subjected to pharmacokinetic analyses using the Osiris Data Warrior program (Figure 3). According to Lipinski’s “Rule of Five”, a compound is considered a good drug candidate for preclinical studies if it meets the following criteria: molecular weight (MW) ≤ 500 Da, rotatable bonds ≤ 10, hydrogen bond acceptors ≤ 10, hydrogen bond donors ≤ 5, and a calculated partition coefficient (cLogP) ≤ 5. For the molecular docking analysis, only compounds with a single violation of Lipinski’s rules were included.

Figure 3A shows the molecular weight of all the compounds obtained from *Physalis angulata* that were analyzed; the compounds that had a molecular weight greater than 500 Da were physagulide P, physagulin A, physagulin F, physagulin I, physalin A, physalin B, physalin D, withaminimin, and withangulatin A. Figure 3B shows the calculated cLogP of each one of the compounds to be evaluated in this study. This parameter allows us to evaluate the hydrophilicity of a drug; therefore, those compounds or drugs that present cLogP values greater than 5 are vulnerable to poor absorption and therefore poor pharmacological action.

The compounds that presented cLogP values greater than 5 were the following: (R)-2-hydroxystearic acid, arachidonic acid, behenic acid, eicosanoic acid, heneicosanoic acid, N-oleoyl-phenylalanine, N-oleyl-leucine, pygenic acid, tricosanoic acid, and myristic acid (Figure 3B). In addition to a high molecular weight and lipophilicity, a compound may reduce its ability to permeate the lipid bilayer or permeate the blood–brain barrier due to the large number of hydrogen donor bonds, hydrogen acceptor bonds, and rotatable bonds [47].

Therefore, ideally, a compound should present ≤ 5 hydrogen donor bonds, ≤10 hydrogen acceptor bonds, and ≤10 rotatable bonds. The compounds extracted from *Physalis angulata* that did not meet these parameters were physalin D, 3-O-caffeoylquinic acid, 5-O-caffeoyl quinic acid, gluconic acid, (R)-2-hydroxystearic acid, arachidonic acid, behenic acid, eicosanoic acid, heneicosanoic acid, N-oleoyl-phenylalanine, N-oleyl-leucine, tricosanoic acid, and myristic acid (Figure 3C–E). 

When performing the pharmacokinetic analysis of the compounds it was evaluated which of these compounds presented more than one violation of Lipiski’s rules, which were then discarded for the molecular docking analysis. Figure 3F shows that the only compounds that presented more than one violation of Lipinski’s rule were (R)-2-hydroxystearic acid, arachidonic acid, behenic acid, eicosanoic acid, heneicosanoic acid, N-oleoyl-phenylalanine, N-oleyl-leucine, physalin D, tricosanoic acid, and myristic acid.

### 3.5. Toxixity Prediction

**Figure 4:**
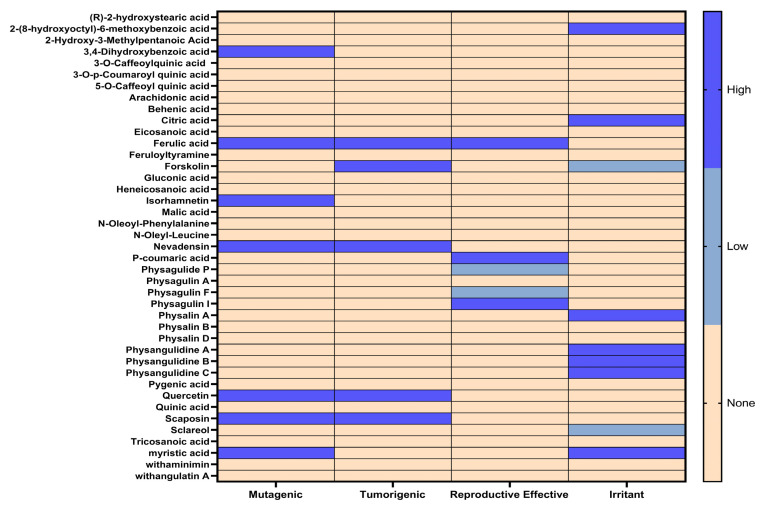
Toxicity risk analysis of compounds obtained from *Physalis angulata* using the Osiris Data warrior software. Note: green color shows low toxic tendency and blue color shows high toxic tendency.

In addition to the pharmacokinetic properties, the toxicological risks of the compounds extracted from *Physalis angulata* were evaluated by in silico analysis using the Osiris Data Warrior computational tool. The toxicological risks that were analyzed in each of the compounds were irritation, tumorigenicity, mutagenicity, and reproductive toxicities (Figure 4). The results obtained from this analysis were represented by a heat map where the results can be analyzed intuitively using a color differential.

Those compounds that presented a high toxicity risk were discarded for molecular docking along with those compounds that did not comply with the pharmacokinetic analysis. The compounds that presented at least a high toxicity risk were 2-(8-hydroxyoctyl)-6-methoxybenzoic acid, 3,4-dihydroxybenzoic acid, citric acid, ferulic acid, forskolin, isorhamnetin, nevadensin, p-coumaric acid, physagulin I, physalin A, physangulidine A, physangulidine B, physangulidine C, quercetin, scaposin, and myristic acid (Figure 4).

## 4. Discussion

### 4.1. Chemical Composition

In the genus *Physalis*, withanolides represent the greatest chemical diversity with more than 350 naturally isolated compounds on record. These compounds exhibit various biological activities, including antifeedant, antimicrobial, immunoregulatory, trypanocidal, leishmanicidal, antidiabetic, antiplasmodial, and anti-inflammatory properties [48,49,50,51], as well as anti-adipogenic effects at the extract level [52] and other biological benefits [53,54,55,56].

Regarding species, *P. angulata* reports the highest number of withanolides [57,58], such as physangulidines A–C, which exhibit antiproliferative effects on tumor cells [59,60,61]. Additionally, derivatives like physalin F have shown immunosuppressive activity in mononuclear cells from patients with HTLV-1-associated myelopathy [62]. Furthermore, studies have identified anti-inflammatory effects mediated by the inhibition of nitric oxide production in macrophages [63,64,65,66].

On the other hand, *P. minima* has also stood out as a rich source of withanolides with anti-inflammatory activity [67], modulating nitric oxide production and the NF-κB pathway in activated macrophages [68,69]. Additionally, new isolates reinforce interest in this species for the development of natural therapies against inflammatory diseases and cancer [70,71]. Likewise, *P. alkekengi* and *P. philadelphica* contain various withanolides with high cytotoxic activity and the regulation of pro-inflammatory factors [72,73].

### 4.2. Antioxidant Properties

The total phenolic content varied considerably among extracts, with ethanolic extracts showing higher values than aqueous extracts in all five organs. The highest value was found in the ethanolic leaf extract (517.49 ± 3.60 mg GA/g), followed by the ethanolic calyx extract (470.57 ± 4.62 mg GA/g). The lowest concentrations were observed in the aqueous fruit extract (77.70 ± 0.88 mg GA/g) and the aqueous root extract (88.98 ± 1.88 mg GA/g). Regarding the DPPH radical scavenging activity and FRAP, values ranged from 24.83 to 199.40 μmol Trolox/g and 66.20 to 476.18 μmol Trolox/g, respectively, with the highest values corresponding to ethanolic leaf extracts and the lowest to aqueous fruit and root extracts, respectively. For ABTS cation radical scavenging, values ranged from 49.39 to 351.24 μmol Trolox/g, with the strongest activity observed in the ethanolic leaf extract, followed by the aqueous leaf extract (174.87 ± 1.96), while the lowest values were found in aqueous root and fruit extracts (52.62 ± 1.02 and 49.39 ± 1.02 μmol Trolox/g, respectively). Comparing each organ of the plant, the ethanolic extracts presented better values than the aqueous extracts, with the ethanolic leaf extract presenting the best values in all the tests and the aqueous fruit extract the lowest.

These findings are consistent with those documented by Ramakrishna et al. [74], where ethanolic leaf extracts exhibited the highest activity in the DPPH assay and the highest total phenolic and flavonoid content compared to fruit extracts. Likewise, *P. angulata* stands out for its potent bioactivity, comparable to species from the genera *Capsicum*, *Solanum*, and *Lycopersicon* [75]. Regarding extract efficiency, evidence supports a higher concentration of bioactive compounds and antioxidant activity in ethanolic extracts of *P. angulata* [76], and enrichment with secondary extracts such as *Moringa oleifera* can further enhance its antioxidant properties [77].

### 4.3. Enzymatic Inhibition

The aqueous fruit extract exhibited AChE inhibition with an IC_50_ of 2 ± 0.05 μg/mL, followed by the ethanolic calyx extract with an IC_50_ of 14 ± 0.06 μg/mL. For BChE, the ethanolic stem extract showed the highest inhibitory activity (IC_50_ = 51 ± 0.06 μg/mL), followed by the aqueous fruit extract (66 ± 0.06 μg/mL). Although the values are higher than the traditional standard, these extracts suggest the potential use of their compounds as neuroprotective agents and for managing cholinergic disorders.

On the other hand, the ethanolic root and stem extracts were the only ones with significant activity against α-glucosidase, with the ethanolic root extract standing out (IC_50_ = 23.487 ± 0.025 μg/mL), followed by the ethanolic stem extract (IC_50_ = 73.559 ± 0.043 μg/mL). Regarding α-amylase inhibition, both ethanolic and aqueous root extracts showed the strongest activity, with IC_50_ values of 101 ± 0.008 μg/mL and 116 ± 0.003 μg/mL, respectively. The combined inhibition of both enzymes suggests that these extracts contain compounds capable of regulating glucose metabolism by modulating carbohydrate digestion and absorption.

The results regarding the inhibition activity of cholinesterase enzymes (AChE and BChE) from *P. angulata* extracts represent one of the first reports and constitute a continuation in the validation of the species pharmacological potential for the future treatment of diseases related to the central nervous system. On the other hand, the antidiabetic activity of *P. angulata* and *P. alkekengi* extracts has been corroborated in both in vitro and in vivo models, with significant suppression of the enzymes α-glucosidase and α-amylase [48,78]. However, our study reports a higher effectiveness of ethanolic and aqueous extracts from some organs of *P. angulata*.

### 4.4. Docking Studies

Compounds obtained from *Physalis angulata* extracts with no more than one violation in their pharmacokinetic properties and without a high toxicity risk, as well as from the known inhibitors galantamine (for acetylcholinesterase and butyrylcholinesterase) and acarbose (for α-amylase and α-glucosidase), were utilized in molecular docking analysis to assess the molecular interactions between the ligands and the amino acid residues responsible for enzymatic inhibition. The most favorable binding energies for each ligand were reported in Kcal/mol and compared to the binding energy of their respective reference inhibitors.

#### 4.4.1. Acetylcholinesterase (TcAChE) Molecular Docking

Table 5 shows the binding affinities of the compounds that were evaluated as possible inhibitors of acetylcholinesterase (TcAChE). It is observed that the compounds that presented a better affinity in the binding site in the acetylcholinesterase enzyme (TcAChE) were physagulin A (−13.6 Kcal/mol), withaminimin (−11.6 Kcal/mol), physalin B (−9.9 Kcal/mol), and feruloyltyramine (−9.7 Kcal/mol); with it being the compounds physagulin A and withaminimin that presented a greater affinity in the acetylcholinesterase (TcAChE) binding site.

**Figure 5:**
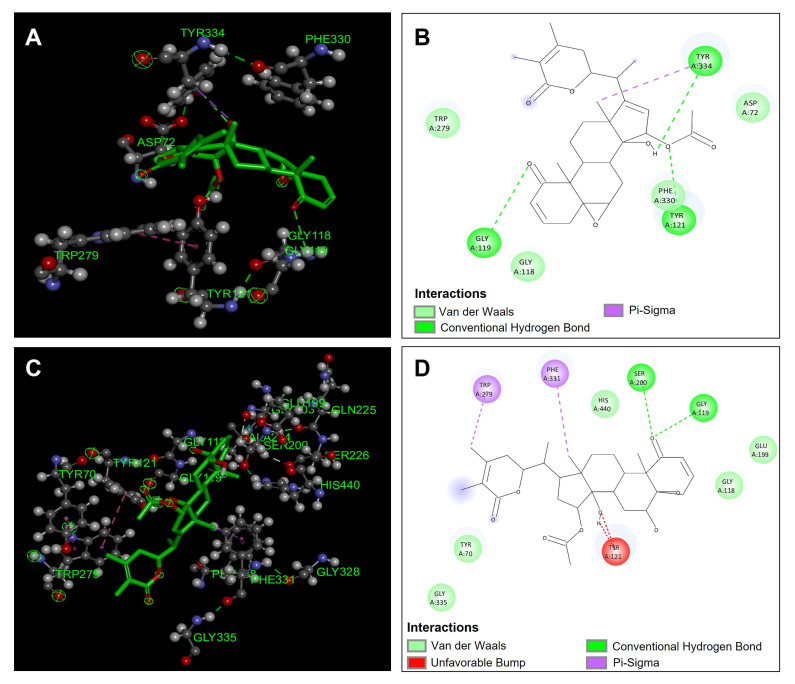
Molecular docking between *Physalis angulata* compounds and acetylcholinesterase (TcAChE). (**A**) Molecular docking of physagulin A and the enzyme acetylcholinesterase (TcAChE); (**B**) predominant molecular interactions of physagulin A and the enzyme acetylcholinesterase (TcAChE); (**C**) molecular docking of withaminimin and the enzyme acetylcholinesterase (TcAChE); (**D**) predominant molecular interactions of withaminimin and the enzyme acetylcholinesterase (TcAChE).

The compound physagulin A has a higher affinity and, therefore, a possible greater inhibition of the acetylcholinesterase enzyme (TcAChE). Physagulin A presented three hydrogen bond type interactions with residues Gly119, Tyr121, and Tyr334, which confirmed significant stability in the binding site in the acetylcholinesterase enzyme (TcAChE) (Figure 5A,B). In addition to hydrogen bond interactions, physagulin A presented four van der Waals type interactions with residues Asp72, Gly118, Trp279, and Phe330, and a π-sigma type interaction with residue Tyr334 (Figure 5B). These interactions made the physagulin A compound present good stability in the binding site; however, it is observed that it did not present direct interactions with the amino acids involved in the inhibition (Ser200 and His440) of the acetylcholinesterase enzyme (TcAChE) (Figure 5A,B). The compound withaminimin was one of the compounds that presented a good affinity in the binding site of the acetylcholinesterase enzyme (TcAChE), although it was not the one that presented the best binding energy. The compound withaminimin presented direct hydrogen bond type interactions with the Ser200 residue and van der Waals type interactions with residue His440; which are both residues involved in the inhibition of acetylcholinesterase (TcAChE) (Figure 5C,D). In addition to the interactions mentioned above, the compound withaminimin presented one more hydrogen bond interaction with the Gly119 residue, and two interactions of π-sigma with residues Trp279 and Phe331 (Figure 5D). The compound withaminimin presented a lower binding energy compared to the compound physagulin A (Table 5) because it presented two unfavorable interactions between the hydroxyl group of withaminimin and the Tyr121 residue (Figure 5C,D).

#### 4.4.2. Butyrylcholinesterase (hBuChE) Molecular Docking

**Figure 6:**
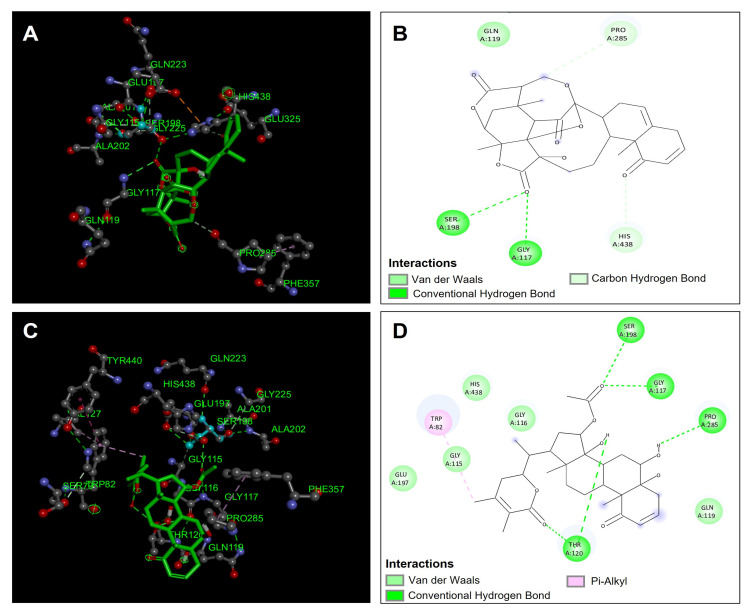
Molecular docking between *Physalis angulata* compounds and butyrylcholinesterase (hBuChE). (**A**) Molecular docking of physalin B and the enzyme butyrylcholinesterase (hBuChE); (**B**) predominant molecular interactions of physalin B and the enzyme butyrylcholinesterase (hBuChE); (**C**) molecular docking of withaminimin and the enzyme butyrylcholinesterase (hBuChE); (**D**) predominant molecular interactions of withaminimin and the enzyme butyrylcholinesterase (hBuChE).

The compounds obtained from *Physalis angulata* were evaluated in the molecular docking analysis to observe the possible inhibition of these compounds against the enzyme butyrylcholinesterase (hBuChE). The compounds were compared with the reference inhibitor galantamine to compare their inhibition against this enzyme. The compounds that presented the best binding affinity were withaminimin (−12.1 kcal/mol), physalin B (−11.6 kcal/mol), pygenic acid (−11.2 kcal/mol), and physagulin F (−11.1 kcal/mol) (Table 5). The compound that presented the best affinity in the butyrylcholinesterase binding site was the compound withaminimin (Figure 6C,D); this compound was one of those that also presented the best binding affinities in acetylcholinesterase. The compound withaminimin presented five hydrogen bond interactions; two interactions took place between the carbonyl group of the ester and residues Ser198 and Gly117 (Figure 6D). These two residues are highly involved in the inhibition of butyrylcholinesterase since they confer important stability within the catalytic site. The other hydrogen bonding interactions occurred between the carbonyl group of the lactone and the hydroxyl group of withaminimin with the residue Thr120. Another hydrogen bond interaction took place between one of the hydroxyl groups of the withaminimin structure with the residue Pro285. In addition to hydrogen bond interactions, the compound withaminimin presented five van der Waals interactions with residues Gly115, Gly116, Gln119, Glu197, and His438; and the compound presents a π -alkyl type interaction between the methyl group of the lactone and the Trp82 residue (Figure 6D). These interactions allowed the compound withaminimin to have good stability and affinity in the catalytic site of butyrylcholinesterase (Figure 6C).

Another compound that presented favorable affinities in the catalytic site of butyrylcholinesterase was the compound physalin B. This compound presented two hydrogen bond type interactions with the amino acids involved in the inhibition of butyrylcholinesterase Ser198 and Gly117 (Figure 6A,B). However, it presents lower affinity compared to withaminimin (Table 5) because it presents fewer hydrogen bond interactions and only presents three van der Waals type interactions (Figure 6B), which, compared to withaminimin (Figure 6D), decreases stability within the catalytic site. Both compounds presented better binding affinities compared to the reference inhibitor galantamine, which makes them good candidate inhibitors of the butyrylcholinesterase enzyme (Table 5).

#### 4.4.3. α-Amylase Molecular Docking

**Figure 7:**
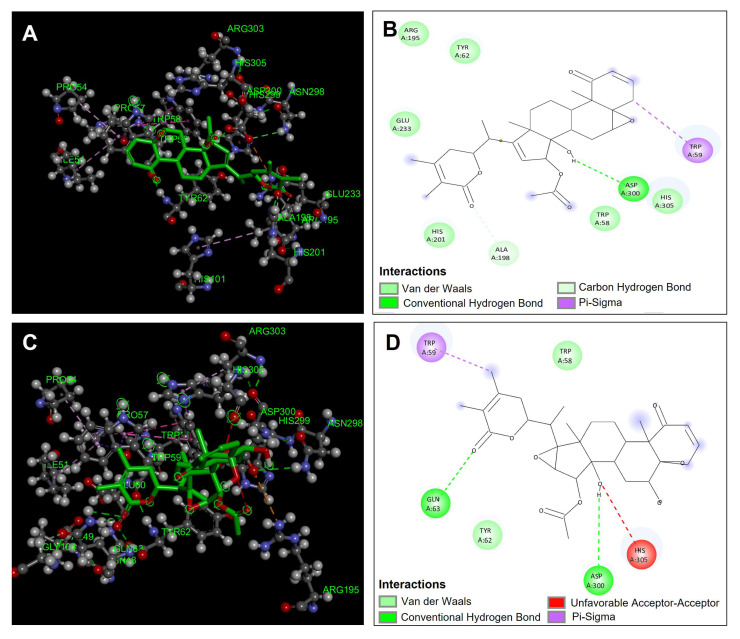
Molecular docking between *Physalis angulata* compounds and α-amylase. (**A**) Molecular docking of physagulin A and the enzyme α-amylase; (**B**) predominant molecular interactions of physagulin A and the enzyme α-amylase; (**C**) molecular docking of physagulin F and the enzyme α-amylase; (**D**) predominant molecular interactions of physagulin F and the enzyme α-amylase.

The compounds obtained from *Physalis angulata* were evaluated and proposed as possible inhibitors of α-amylase, which were evaluated by molecular docking. The compounds that presented the best affinities in the catalytic site of the enzyme were physa- gulin A (−11.3 kcal/mol), physagulin F (−10.1 kcal/mol), pygenic acid (−9.8 kcal/mol), and physalin B (−9.7 kcal /mol) (Table 5). Figure 7A,B show the predominant interactions of the compound physagulin A, which was the one that presented the best binding affinity and therefore better inhibition of α-amylase. Physagulin A presented a hydrogen bond interaction with one of the amino acids directly involved in the inhibition of α-amylase (Asp300) (Figure 7A,B). Also, six van de Waals interactions were observed with residues Trp58, Tyr62, Arg195, His201, Glu233, and His305. A π-sigma type interaction was observed between the sigma electrons of the methylene group of physagulin A and the π electrons of the Trp59 residue (Figure 7B).

Another of the compounds that presented favorable binding affinity was the compound physagulin F. This compound showed two hydrogen bond interactions with residues Gln63 and Asp300 which are important interactions for carrying out the inhibition of α-amylase and interactions that give it considerable stability to the compound at the catalytic site. However, although the physagulin F compound presented greater hydrogen bond interactions, it had a lower binding affinity compared to the physagulin A compound that only presented one hydrogen bond interaction (Asp300). This is mainly due to the fact that the physagulin F compound presented an unfavorable acceptor–acceptor interaction between the hydroxyl group and the His305 residue, which gives it a small geometric instability within the binding site, unlike the physagulin A compound (Figure 7C,D).

#### 4.4.4. α-Glucosidase Molecular Docking

**Figure 8:**
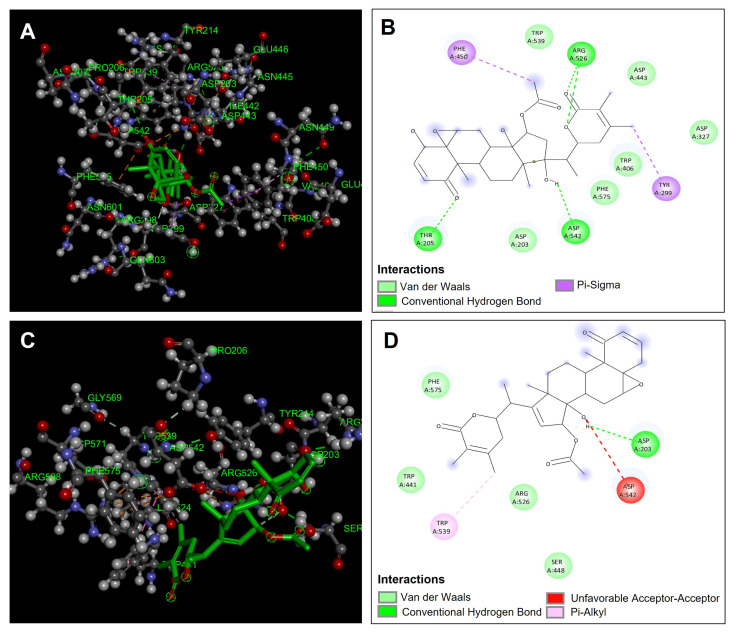
Molecular docking between *Physalis angulata* compounds and α-glucosidase. (**A**) Molecular docking of physagulide P and the enzyme α-glucosidase; (**B**) predominant molecular interactions of physagulide P and the enzyme α-glucosidase; (**C**) molecular docking of physagulin A and the enzyme α-glucosidase; (**D**) predominant molecular interactions of physagulin A and the enzyme α-glucosidase.

The results of the in silico analysis of the compounds obtained from *Physalis angulata* were evaluated against the enzyme α-glucosidase, obtaining the binding affinities in each of the compounds (Table 5). The compounds were compared with the reference inhibitor acarbose to observe if the compounds had the same or better performance than the reference inhibitor. The results showed that the compounds physagulide P (−9.2 kcal/mol), physagulin A (−8.8 kcal/mol), pygenic acid (−8.6 kcal/mol), and withaminimin (−8.4 kcal/mol) were the ones that presented the best results of binding affinity and therefore inhibition against the enzyme α-glucosidase. It is observed that all these compounds presented a better binding affinity in comparison with the reference inhibitor acarbose. Figure 8A,B show the geometric conformation and the predominant interactions of the physagulide P compound in the catalytic site. The compound physagulide P showed four hydrogen bond interactions; I present two interactions with residue Arg546 and the other two interactions I carry out with residues Thr205 and Asp542 (Figure 8B). Also, it presented two π-sigma interactions with residues Tyr299 and Phe450, and six van der Waals interactions with residues Asp203, Asp327, Trp406, Asp443, Trp539, and Phe575 (Figure 8A,B). All these interactions conferred greater genomic stability within the binding site, allowing its binding affinity to be favorable for possible inhibition.

The compound physagulin A was again one of the compounds that presented the highest binding affinities. This compound presented only a hydrogen bond interaction between the hydroxyl group and the Asp203 residue. Also, a π-alkyl type interaction was presented with residue Trp539 and four van der Waals type interactions with residues Trp441, Ser448, Arg526, and Phe575 (Figure 8C,D). This compound had a lower behavior compared to the physagulide P compound because it presented a favorable acceptor–acceptor interaction between the hydroxyl group of its structure and the Asp542 residue, which causes its stability to be lower compared to physagulide P (Figure 8D).

## 5. Conclusions

In summary, the findings of this study reinforce the scientific evidence on the pharmacological potential of *P. angulata* and its relevance as a medicinal and ethnobotanical resource, whilst also being the first study report about the chemical composition and biological activity for this species collected in the Peruvian Amazon. The chemical characterization using UHPLC-ESI-QTOF-MS enabled the identification of 42 compounds in aqueous and ethanolic extracts from different plant organs, highlighting the presence of physalins and withanolides, known for their multiple biological activities. From a functional perspective, the leaf and fruit extracts demonstrated remarkable antioxidant capacity in the FRAP, DPPH, and ABTS assays, suggesting their potential as protective agents against oxidative stress, a key factor in various chronic and degenerative diseases. Similarly, the calyx and fruit extracts showed greater effectiveness in inhibiting the AChE, BChE, α-glucosidase, and α-amylase enzymes, indicating possible applications in the treatment of neurodegenerative diseases and metabolic disorders such as type 2 diabetes. The in silico analysis provided deeper insights into the pharmacokinetic properties of the identified compounds and their interactions with the catalytic sites of the studied enzymes, further supporting their therapeutic potential. Altogether, these findings open new perspectives for the development of phytopharmaceuticals with applications in various diseases. Nevertheless, further in vivo studies and clinical trials are necessary to confirm the efficacy and safety of the extracts and their main compounds, as well as to clarify their molecular mechanisms of action.

## Figures and Tables

**Table 1:** Grid box parameters for docking for acetylcholinesterase (TcAChE), butyrylcholinesterase (hBChE), α-amylase, and α-glucosidase.

Enzymes	Grid Box Size (Å)			Grid Center Coordinate		
	X	Y	Z	X	Y	Z
Acetylcholinesterase	30	30	30	3.67	65.99	64.08
Butyrylcholinesterase	30	30	30	134.11	115.17	38.13
α-Amylase	50	50	50	12.37	48.13	26.24
α-Glucosidase	40	40	40	−20.83	−6.56	−5.04

**Table 2:** Identification by UHPLC-QToF-MS-MS of metabolites present in both ethanolic and aqueous extracts of *Physalis angulata*.

Peak	Retention Time (min)	UV Max	Tentative Identification	[M − H]^−^	Measured Mass (*m*/*z*)	Theoretical Mass (*m*/*z*)	Accuracy (ppm)	MS Ions (ppm)
1	0.45	-	Na formiate (internal standard)	C_4_H_2_O_4_	112.9829	112.9856	3.1	-
2	0.82	273	p-Coumaric acid	C_9_H_8_O_3_	164.04706	163.03978	−2.36	-
3	0.98	227-272	Gluconic acid	C_6_H_12_O_7_	195.05058	195.04993	3.351	-
4	3.43	229-274	Malic acid	C_4_H_6_O_5_	133.01355	133.01315	3.008	-
5	4.54	227	Citric acid	C_6_H_8_O_7_	191.01933	191.01863	3.684	-
6	4.86	273	Quinic acid	C_7_H_12_O_6_	192.06287	191.05559	−1.37	-
7	5.07	227-265	3,4-Dihydroxybenzoic acid (protocatechuic acid)	C_7_H_6_O_4_	154.02654	153.01927	2.987	-
8	5.16	196-227	2-(8-Hydroxyoctyl)-6-methoxybenzoic acid	C_16_H_24_O_4_	280.16649	279.15922	−3.46	-
9	5.24	196-204-269	Feruloyltyramine	C_18_H_19_NO_4_	313.13105	312.12377	−1.07	-
10	5.54	227-257-312	3-O-Caffeoylquinic acid *	C_16_H_17_O_9_^−^	353.08774	353.08671	2.916	-
11	6.24	227-257-312	Forskolin	C_22_H_34_O_7_	410.22992	409.22264	−1.16	179.78584, 160.84163, 135.04446
12	6.43	248-270-332	Physalin B	C_27_H_30_O_15_	509.18128	510.18856	0.906	-
13	7.16	214-280-311	Ferulic acid *	C_10_H_10_O_4_	193.05020	193.04954	3.450	-
14	7.25	214-280-312	3-O-*p*-Coumaroylquinic acid	C_16_H_18_O_8_	337.09283	337.09179	3.087	191.14720, 172.27388, 162.83879, 119.16192
15	7.52	227-280-309	Scaposin	C_19_H_18_O_9_	390.09509	389.08781	0.23	-
16	8.12	-	Nevadensin	C_18_H_16_O_7_	344.08961	343.08233	1.75	-
17	8.25	227-257-310	5-O-Caffeoylquinic acid	C_16_H_18_O_9_	353.08783	353.08671	3.175	191.05560, 135.04462, 133.99362
18	8.53	227-257-311	Physalin A	C_28_H_30_O_10_	525.17576	526.18303	2.721	242.81560, 191.05563
19	8.75	1.3	2-Hydroxy-3-methylpentanoic acid	C_6_H_12_O_3_	131.07141	132.07868	0.298	-
20	9.43	250	Physagulin I	C_30_H_39_ClO_8_	562.31525	563.32252	2.828	-
21	9.86	249-283	Physagulin F	C_30_H_40_O_9_	543.22105	544.22832	3.558	-
22	10.12	251-304-329	Physangulidine B	C_28_H_36_O_8_	499.23371	500.24099	0.189	255.02881, 151.06294
23	10.54	249-264-334	Physalin D	C_28_H_32_O_11_	543.18735	544.19463	2.941	-
24	10.63	249-283-324	Eicosanoic acid	C_20_H_40_O_2_	312.30296	311.29569	−2.11	-
25	11.12	248-267-334	Myristic acid	C_14_H_28_O_2_	228.20857	227.20133	2.517	-
26	11.53	255-300-351	Physagulide P	C_30_H_40_O_9_	545.3105	546.31778	2.890	385.14127, 300.02771
27	12.01	253-288-311	Withangulatin A	C_30_H_38_O_8_	525.17605	526.18329	2.811	284.30374, 125.87235
28	12.54	-	Arachidonic acid	C_2_0H_32_O_2_	304.24051	303.23323	0.34	-
29	12.67	256-348	Withaminimin	C_30_H_40_O_8_	527.21404	528.22132	2.948	-
30	13.21	251-288-332	Physangulidine A	C_28_H_36_O_8_	499.23371	500.24099	2.607	357.40594, 327.08704, 285.13358
31	13.26	-	Pygenic acid	C_30_H_48_O_4_	472.35429	471.34701	−2.05	-
32	13.63	252-274-281	Physangulidine C	C_28_H_35_O_8_	499.25377	500.24353	−12,455	298.55157, 287.13138
33	14.23	251-281	Physagulin A	C_30_H_38_O_7_	509.23287	510.24015	2.473	-
34	14.76	-	(R)-2-Hydroxystearic acid	C_18_H_36_O_3_	300.26636	299.25908	−0.29	-
35	15.12	-	N-Oleoyl-phenylalanine	C_27_H_43_NO_3_	429.32436	428.31709	0.36	-
36	15.23	250-281-311	N-Oleyl-leucine	C_24_H_45_NO_3_	395.33967	394.33239	−0.72	-
37	15.54	254-290-311	Heneicosanoic acid	C_21_H_42_O_2_	326.31784	325.31056	−1.98	-
38	16.26	253-292-311	Tricosanoic acid	C_23_H_46_O_2_	354.34983	353.34256	−1.63	-
39	16.57	251-267-280	Behenic acid	C_22_H_44_O_2_	340.33361	339.32633	−2.53	215.00941
40	16.52	252-274-281	Physangulidine C	C_18_H_22_O_5_	499.25377	500.24353	−12,455	298.55157, 287.13138
41	17.57	251-311	Quercetin *	C_15_H_10_O_7_	301.03549	301.03428	4.029	284.31473, 151.00281
42	18.12	252-288-304	Isorhamnetin *	C_16_H_12_O_7_	315.05096	315.04993	3.286	270.46667, 151.20018, 108.47343, 107.73669
43	19.23	245-285-311	Sclareol	C_20_H_36_O_2_	308.27115	307.26387	−0.09	-

* Identified by co-spiking experiments using authentic standard compounds.

**Table 3:** Total phenolic content (TPC) and antioxidant activity from *Physalis angulata* extracts.

Extract/Assay	TPC (mg GA/g)	DPPH (μmoL Trolox/g)	ABTS (μmoL Trolox/g)	FRAP (μmoL Trolox/g)
Aqueous root	88.98 ± 1.88 ^a^	26.71 ± 0.96 ^a^	52.62 ± 1.02 ^a^	66.20 ± 1.26 ^a^
Aqueous stem	90.52 ± 1.17 ^a^	26.27 ± 1.00 ^a^	55.37 ± 1.05 ^a^	73.33 ± 1.19 ^b^
Aqueous leaves	349.44 ± 2.53 ^b^	83.67 ± 0.83 ^b^	174.87 ± 1.96 ^b^	137.44 ± 2.16 ^c^
Aqueous calyx	182.54 ± 1.29 ^c^	53.47 ± 1.02 ^c^	104.42 ± 1.39 ^c^	121.63 ± 2.32 ^d^
Aqueous fruit	77.70 ± 0.88 ^d^	24.83 ± 1.03 ^a^	55.08 ± 0.96 ^a^	66.70 ± 0.93 ^a^
Ethanolic root	268.55 ± 2.00 ^e^	47.73 ± 0.98 ^d^	86.43 ± 1.29 ^d^	116.15 ± 1.12 ^d^
Ethanolic stem	177.18 ± 1.31 ^c^	50.90 ± 0.96 ^cd^	93.86 ± 1.93 ^e^	132.43 ± 2.01 ^c^
Ethanolic leaves	517.49 ± 3.60 ^f^	199.40 ± 1.98 ^e^	351.24 ± 5.18 ^f^	476.18 ± 3.72 ^e^
Ethanolic calyx	470.57 ± 4.62 ^g^	145.79 ± 2.51 ^f^	153.14 ± 1.94 ^g^	374.41 ± 4.38 ^f^
Ethanolic fruit	145.36 ± 2.23 ^h^	44.96 ± 1.00 ^g^	49.39 ± 1.02 ^a^	115.36 ± 1.25 ^d^

Values assigned different letters on the same column indicate are significant difference at 0.05 level of significance according to Tukey’s test.

**Table 4:** Enzyme inhibitory activity from *Physalis angulata* extracts.

Extract/Assay	AChE IC_50_ (µg/mL)	BChE IC_50_ (µg/mL)	α-Glucosidase IC_50_ (µg/mL)	α-Amylase IC_50_ (µg/mL)
Aqueous root	46 ± 0.06 ^a^	205 ± 0.08 ^a^	1284.249 ± 0.045 ^a^	116 ± 0.003 ^a^
Aqueous stem	47 ± 0.06 ^a^	70 ± 0.08 ^b^	ND	125 ± 0.003 ^b^
Aqueous leaves	68 ± 0.09 ^b^	86 ± 0.09 ^c^	ND	197 ± 0.003 ^c^
Aqueous calyx	34 ± 0.08 ^c^	93 ± 0.09 ^c^	ND	197 ± 0.007 ^c^
Aqueous fruit	2 ± 0.05 ^d^	66 ± 0.06 ^b^	ND	297 ± 0.006 ^d^
Ethanolic root	43 ± 0.05 ^a^	78 ± 0.09 ^d^	23.487 ± 0.025 ^b^	101 ± 0.008 ^e^
Ethanolic stem	27 ± 0.05 ^e^	51 ± 0.06 ^e^	73.559 ± 0.043 ^c^	150 ± 0.005 ^f^
Ethanolic leaves	34 ± 0.06 ^c^	67 ± 0.10 ^b^	ND	412 ± 0.003 ^g^
Ethanolic calyx	14 ± 0.06 ^f^	72 ± 0.05 ^b^	ND	120 ± 0.005 ^b^
Ethanolic fruit	31 ± 0.04 ^c^	98 ± 0.06 ^f^	ND	122 ± 0.005 ^b^
Galantamine ^#^	0.266 ± 0.029 ^g^	3.824 ± 0.024 ^g^	-	-
Acarbose ^#^	-	-	229.412 ± 0.031 ^d^	6.477 ± 0.003 ^h^

Values assigned different letters on the same column indicate are significant difference at 0.05 level of significance according to Tukey’s test. # Positive control. AChE: acetylcholinesterase; BChE: butyrylcholinesterase; ND: not detected.

**Table 5:** Binding affinities resulting from molecular docking of phytochemicals selected in the extracts from *Physalis angulata* on acetylcholinesterase (TcAChE), butyrylcholinesterase (hBChE), α-amylase, and α-glucosidase.

Compounds	Acetylcholinesterase (TcAChE) (Kcal/mol)	Butyrylcholinesterase (hBChE) (Kcal/mol)	α-Amylase (Kcal/mol)	α-Glucosidase (Kcal/mol)
2-Hydroxy-3-methylpentanoic acid	−5.4	−5.2	−4.5	−5.4
3-O-Caffeoylquinic acid	−9.2	−9.1	−7.9	−6.9
3-O-p-Coumaroyl quinic acid	−9.4	−8.7	−7.9	−6.9
5-O-Caffeoyl quinic acid	−9.2	−8.3	−7.7	−7.1
Feruloyltyramine	−9.7	−8.6	−7.9	−7.2
Gluconic acid	−5.3	−5.4	−6.1	−5.1
Malic acid	−4.6	−5.1	−4.6	−4.7
Physagulide P	−9.2	−10.4	−8.7	−9.2
Physagulin A	−13.6	−10.8	−11.3	−8.8
Physagulin F	−8.5	−11.1	−10.1	−8.1
Physalin B	−9.9	−11.6	−9.7	−8.2
Pygenic acid	−9.1	−11.2	−9.8	−8.6
Quinic acid	−6.1	−6.1	−6.3	−5.5
Sclareol	−9.1	−9.2	−7.5	−6.8
Withaminimin	−11.6	−12.1	−9.1	−8.4
Withangulatin A	−1.1	−1.2	−1.1	−1.3
Galanthamine *	−11.30	−9.20	--	--
Acarbose *	--	--	−8.10	−7.50

Note: * reference inhibitor.

## Data Availability

The data presented in this study are available on request from the corresponding authors.
